# Applying Deep Learning to Accelerated Clinical Brain Magnetic Resonance Imaging for Multiple Sclerosis

**DOI:** 10.3389/fneur.2021.685276

**Published:** 2021-09-27

**Authors:** Ashika Mani, Tales Santini, Radhika Puppala, Megan Dahl, Shruthi Venkatesh, Elizabeth Walker, Megan DeHaven, Cigdem Isitan, Tamer S. Ibrahim, Long Wang, Tao Zhang, Enhao Gong, Jessica Barrios-Martinez, Fang-Cheng Yeh, Robert Krafty, Joseph M. Mettenburg, Zongqi Xia

**Affiliations:** ^1^Department of Biostatistics, University of Pittsburgh, Pittsburgh, PA, United States; ^2^Department of Bioengineering, University of Pittsburgh, Pittsburgh, PA, United States; ^3^Department of Neurology, University of Pittsburgh, Pittsburgh, PA, United States; ^4^Subtle Medical Inc., Menlo Park, CA, United States; ^5^Department of Neurological Surgery, University of Pittsburgh, Pittsburgh, PA, United States; ^6^Department of Biostatistics and Bioinformatics, Emory University, Atlanta, GA, United States; ^7^Department of Radiology, University of Pittsburgh, Pittsburgh, PA, United States

**Keywords:** multiple sclerosis, deep learning, artificial intelligence, magnetic resonance imaging, accelerated acquisition, patient-reported outcome (PRO), brain volume, DBPN

## Abstract

**Background:** Magnetic resonance (MR) scans are routine clinical procedures for monitoring people with multiple sclerosis (PwMS). Patient discomfort, timely scheduling, and financial burden motivate the need to accelerate MR scan time. We examined the clinical application of a deep learning (DL) model in restoring the image quality of accelerated routine clinical brain MR scans for PwMS.

**Methods:** We acquired fast 3D T1w BRAVO and fast 3D T2w FLAIR MRI sequences (half the phase encodes and half the number of slices) in parallel to conventional parameters. Using a subset of the scans, we trained a DL model to generate images from fast scans with quality similar to the conventional scans and then applied the model to the remaining scans. We calculated clinically relevant T1w volumetrics (normalized whole brain, thalamic, gray matter, and white matter volume) for all scans and T2 lesion volume in a sub-analysis. We performed paired *t*-tests comparing conventional, fast, and fast with DL for these volumetrics, and fit repeated measures mixed-effects models to test for differences in correlations between volumetrics and clinically relevant patient-reported outcomes (PRO).

**Results:** We found statistically significant but small differences between conventional and fast scans with DL for all T1w volumetrics. There was no difference in the extent to which the key T1w volumetrics correlated with clinically relevant PROs of MS symptom burden and neurological disability.

**Conclusion:** A deep learning model that improves the image quality of the accelerated routine clinical brain MR scans has the potential to inform clinically relevant outcomes in MS.

## Introduction

Routine magnetic resonance (MR) scans are the standard of care for monitoring disease activity and progression in people with multiple sclerosis (PwMS) (1). Most PwMS undergo brain MR scans yearly, with individual factors such as changes in disease activity and disease-modifying treatment (DMT) altering the scan frequency. Prolonged MR scan time contributes to patient discomfort, poor image quality due to motion, delays in scheduling, potentially high medical cost, and financial burden for PwMS (2). Thus, accelerating the acquisition time of clinical MR scans could benefit PwMS and generally improve access to critical diagnostic imaging. However, diminished image quality (e.g., contrast to noise ratio, resolution) is the main challenge preventing the clinical application of MR scans with accelerated acquisition time.

Artificial intelligence (AI) approaches could potentially address the loss of MR image quality in accelerated scans. Deep learning (DL) models such as convolutional neural networks (CNNs) enhance MR image quality of the fast scans without compromising relevant image information passed through each layer (3–6). Compared to supervised learning algorithms, CNNs show comparable aptitude and often greater adaptability in MRI post-processing (7). Deep back-projection network (DBPN) is a class of CNN that outperforms other methods given its ability to self-correct errors using back-projection (8).

In this study, we evaluated the clinical application of a DL model based on DBPN that employed noise-reducing and sharpness-enhancing functions. Specifically, we assessed whether the DL model improved the quality of fast clinical brain MR images acquired with accelerated time and whether the key volumetrics from brain MR preserved their correlations with clinically relevant neurological outcomes to the extent comparable to the benchmark conventional MR scans. Here, we prioritized T1-weighted volumetrics (i.e., whole brain, gray matter, white matter, thalami) that are known to be associated with subsequent clinical outcomes in MS (9–18).

## Methods

### Data Source

We recruited participants from a clinic-based, prospective MS cohort study (Prospective Investigation of Multiple Sclerosis in the Three Rivers Region, PROMOTE) based in the Pittsburgh region (PA, USA). The Institutional Review Board of the University of Pittsburgh approved this study. All participants completed the informed consent process.

### MRI Acquisition

One hundred and fifteen participants underwent routine clinical brain MR studies on a GE Discovery MR750 3-Tesla scanner between September 2018 and January 2020, some completing multiple scans on separate days. In addition to the institutional clinical protocol that included the standard (or conventional) 3D T1w BRAVO (FE/PE/SE: 220 × 220 × 126, scan time 2:57), 3D T2 FLAIR (FE/PE/SE: 256 × 224 × 240, scan time 6:40), and other routine clinical sequences, we acquired an accelerated (or fast) 3D T1w BRAVO (FE/PE/SE: 220 × 128 × 64, scan time 1:13) and an accelerated (or fast) 3D T2w FLAIR (FE/PE/SE: 256 × 128 × 120, scan time 2:17) during the same MR exam. Compared to the conventional T1w BRAVO and T2w FLAIR, the fast sequences were accelerated by a factor of 2 in both the phase and slice directions.

### Deep Learning

We developed a DL model based on DBPN (8) to enhance the image quality for the fast sequences from clinical brain MR scans. The DL model input the fast sequences and generated high resolution images similar to that of the conventional sequences. The output of the DL model had twice the slice number as that of the input. To incorporate the slice information, we applied a 2.5 D model with five adjacent slices. We trained the DL model with the first 15 randomly selected scans, with images from the conventional sequences serving as the ground truth. An L1 loss was applied in training to measure the difference between the DL output and ground truth. We applied image pre-processing, including image registration (19), bias field correction (20), and image normalization to the training data. We implemented the DL model in TensorFlow and trained on an NVIDIA V100 GPU with an ADAM optimizer (21). After excluding the 15 training scans and 7 scans acquired with the incorrect conventional and/or fast sequences, we applied the DL model to the remaining 108 scans for evaluation.

### MR Image Analysis

**Figure 1:**
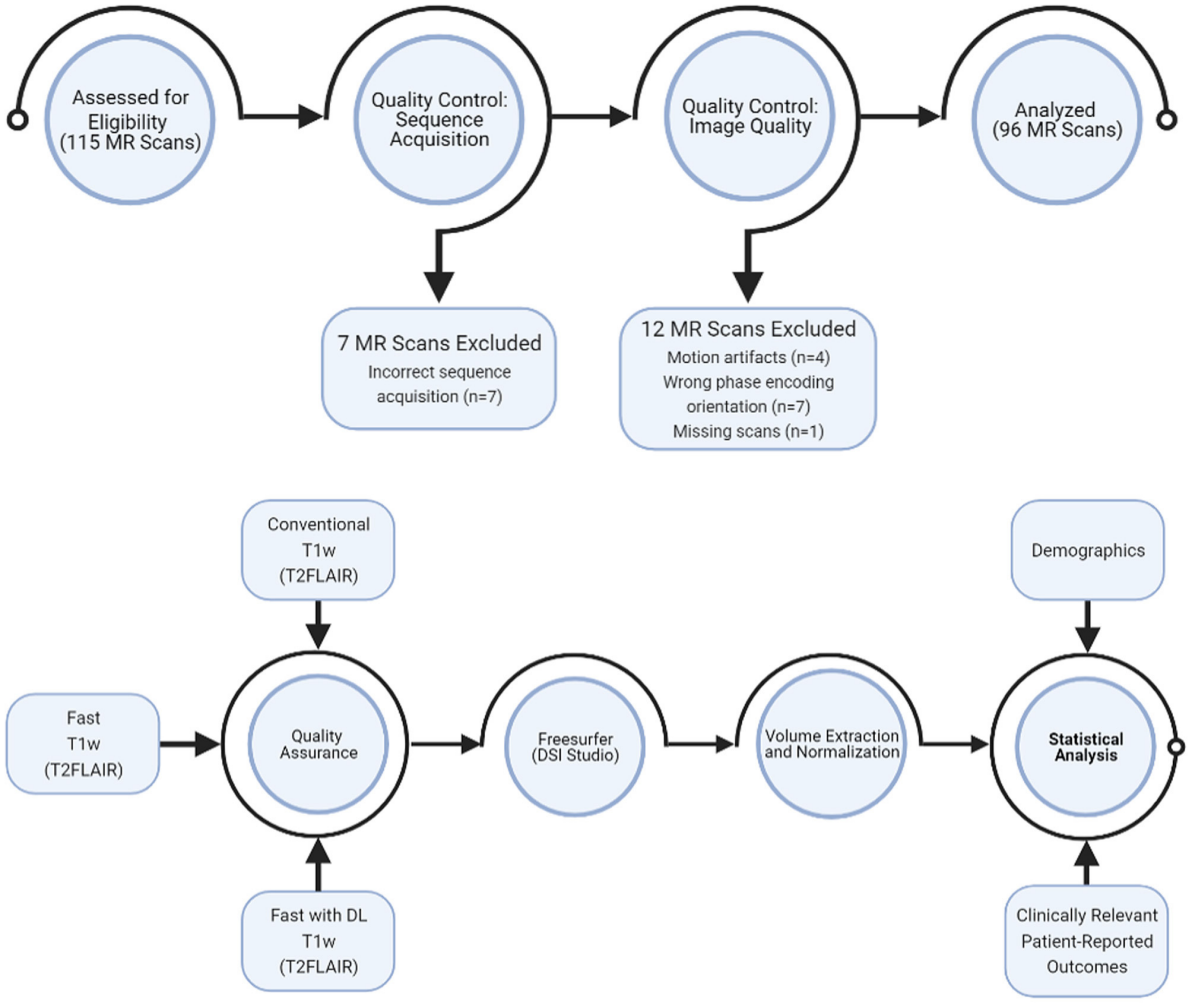
Study workflow.

Figure 1 showed the overall workflow. For this study, we prioritized T1w volumetric analysis. As quality control, we first examined the raw T1w images to remove scan with: (1) excessive motion artifacts that could decrease the automatic segmentation accuracy (4 out of 108); (2) acquisitions in the wrong phase encoding orientation (7 out of 108); and (3) missing scan (1 out of 108). Using the FreeSurfer software version 6.0 (http://surfer.nmr.mgh.harvard.edu/) (22), we then computed the volumes of 96 sets of T1w MR images, including the following regions: total brain, total thalamus, total cerebral gray matter, and total cerebral white matter as well as intracranial space. These regions were chosen based on known correlation with clinical outcomes in MS (9–15, 17, 18). We extracted the volumes from the automatic segmentation file “aseg” of FreeSurfer and normalized each volume measure by the intracranial volume in each individual. Normalized volumes had no unit.

For exploratory analysis, we manually delineated the T2 lesions and calculated the T2 lesion volume in a subset of 30 MR scans using DSI Studio (http://dsi-studio.labsolver.org) (23). Two initial raters (RP and MD) performed the manual correction, and a third supervising rater (CI) performed the final manual correction. We first loaded the T2 FLAIR images in DSI Studio from Step T3. The lesion drawing task began with placing multiple 3D spheres to cover each of the T2 hyperintense regions. DSI Studio provides a 3D interactive function to place and move spheres in the 3D space to complete this task quickly. The raters then refined the contours of the lesions by applying an intensity threshold. The exact value of the threshold was adjustable to achieve the best segmentation results. Each scan required an average of 15–30 min to complete the manual correction.

### Patient-Reported Outcomes

To assess the clinical relevance of the neuroimaging measures, we used two clinically relevant patient-reported outcomes of neurological function validated in PwMS. First, the Multiple Sclerosis Rating Scale-Revised (MSRS-R) assessed the MS symptom burden across eight domains: walking, using arms and hands, vision, speech, swallowing, cognition, sensation, and bowel and bladder control (24, 25). Each domain included a sub-score ranging from 0 (no symptoms) to 4 (severe disability) for a maximum total score of 32. Second, Patient Determined Disease Steps (PDDS) assessed the gait impairment, ranging from 0 (normal gait) to 8 (bedridden). We categorized patients as having severe disabilities based on consistent requirements for assistive devices for distances longer than 25 feet (PDDS of 4). This threshold approximated the clinician-rated Extended Disability Status Scale (EDSS) score of 6 (26).

### Statistical Analysis

All analyses were completed using R version 4.0.3 (27). For the paired *t-*tests, a two-sided *P* < 0.0125 was indicative of statistical significance as we used the Bonferroni Correction method to obtain this significance level (0.05/4 = 0.0125), given the four different T1w volumetrics being tested. For descriptive variables, we expressed continuous data as mean and standard deviation (SD) or medians and interquartile ranges, and categorical data as frequencies and percentages.

We first performed paired *t*-tests to compare the three types of MR acquisition (conventional, fast, fast with DL) for the four T1w volume measures: normalized brain volume (NBV), normalized thalamic volume (NThV), normalized gray matter volume (NGMV), and normalized white matter volume (NWMV).

To measure the association between the four T1w volumetrics (NBV, NThV, NGMV, NWMV) and the two patient-reported clinical outcomes (MSRS-R, PDDS), we developed a repeated measures multivariate linear mixed-effects model for T1w volumetrics that included the type of MR acquisition conducted as the fixed between-subject factor, a random subject effect and clinical outcomes (see Supplementary Material for formula). We included interaction terms between the clinical outcomes tested and the type of MR acquisition, with conventional MR acquisition as the reference point or benchmark. The interaction terms quantified (1) differences in associations with clinical outcomes between fast scans and conventional scans, and (2) differences in associations with clinical outcomes between fast scans with DL and conventional scans. To account for the contribution of age, sex, race, ethnicity, disease duration in years, clinical type (e.g., relapsing), and DMT (i.e., treatment) status on the clinical outcomes, we adjusted these covariates as fixed effects in the model. We performed exploratory analysis examining the multivariate association between T2 lesion volume and clinical outcomes.

### Data and Code Availability

Code for analysis and figure generation is available at: https://github.com/ashikamani/MS-MRI-. Anonymous data that support the findings of this study are available upon reasonable request to the corresponding author.

## Results

### Participant Demographics

This study includes 87 unique PwMS (see Table 1 for demographics). After excluding training scans and evaluation scans with quality control failure, there were a total of 96 MR scans for evaluation. Nine patients had two MR scans occurring on separate days. The mean age of the participants was 47 years. Most participants were women and of Non-Hispanic European descent (70.2 and 80.5%, respectively). Most participants (86.2%) had the relapsing type of MS with mostly mild physical disability and gait impairment (mean PDDS of 1.7) and mild MS symptom burden (median MSRS-R of 4). The mean disease duration (i.e., the interval between the date of the participant's first neurological symptoms and the date of MR scan) was 15 years. At the time of the MR, most participants (72.4%) received DMT with 5.7% on high-efficacy treatment.

**Table 1:** Characteristics of the study participants.

**Characteristics**	**Participants (*N* = 87)^b^**
Age (years), Mean ± Standard deviation	46.8 ± 13.3
Men, *n* (%)	26 (29.8)
European-descent, *n* (%)	73 (83.9)
Non-hispanic, *n* (%)	84 (96.6)
Non-hispanic European descent, *n* (%)	70 (80.5)
PDDS^a^, Mean ± SD	1.7 ± 1.8
MSRS-R^a^, median (interquartile range)	4 (2–9)
RMS^a^, *n* (%)	75 (86.2)
Disease duration (years), Mean ± SD	14.9 ± 19.3
No treatment, *n* (%)	24 (27.6)
High-efficacy treatment, *n* (%)	5 (5.7)

a *PDDS, patient-determined disease steps; MSRS-R, multiple sclerosis rating scale-revised; RMS: relapsing clinical type*.

b *Because nine participants had two MR scans on separate days, the total number of participants was 87, whereas the total number of MR scans (occurring on separate days) for evaluation was 96*.

### Comparison of MR Acquisition Methods for T1w MRI Volumes

**Figure 2:**
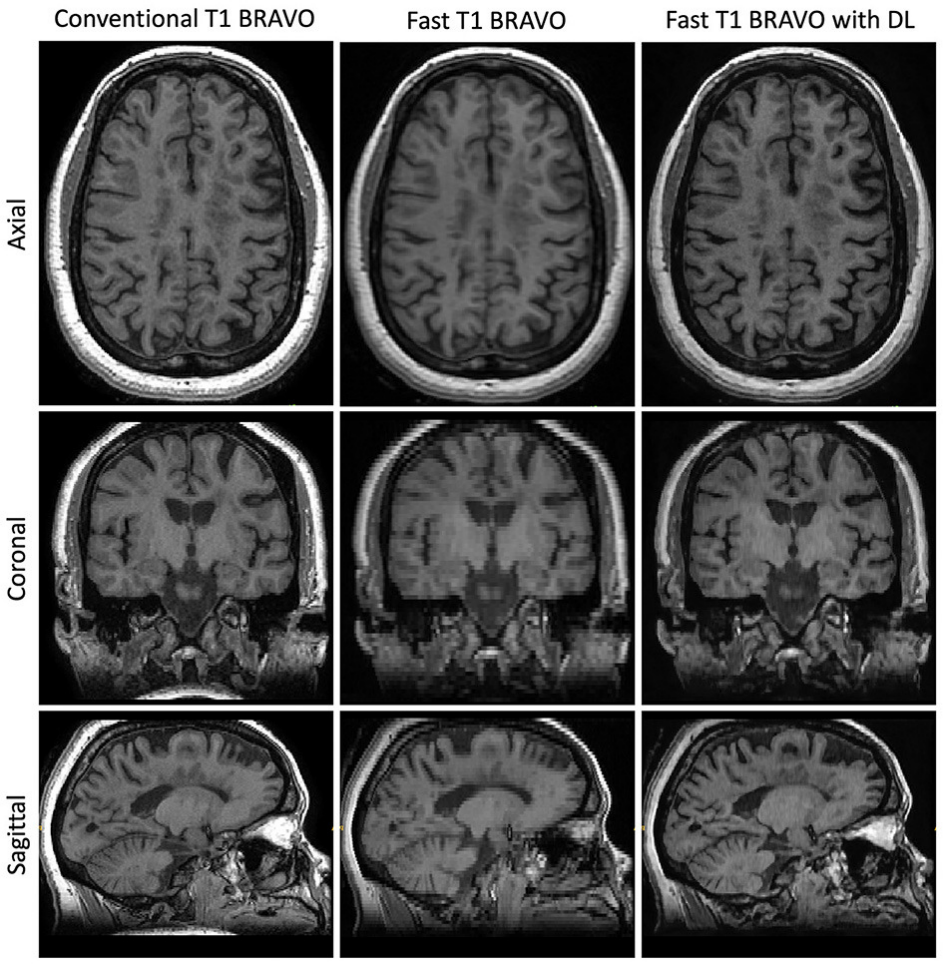
Representative T1w images. Central slices in a similar position for the acquisitions using the three methods: Conventional T1w BRAVO (2:57 min total acquisition time, 220 × 220 × 126 matrix size), fast T1w BRAVO (1:13 min total acquisition time, 220 × 128 × 64 matrix size), and fast T1w BRAVO with DL (same acquisition time and matrix size as the fast T1w BRAVO). BRAVO is the T1-weighted sequence for brain volume imaging on GE MR scanner.

**Figure 3:**
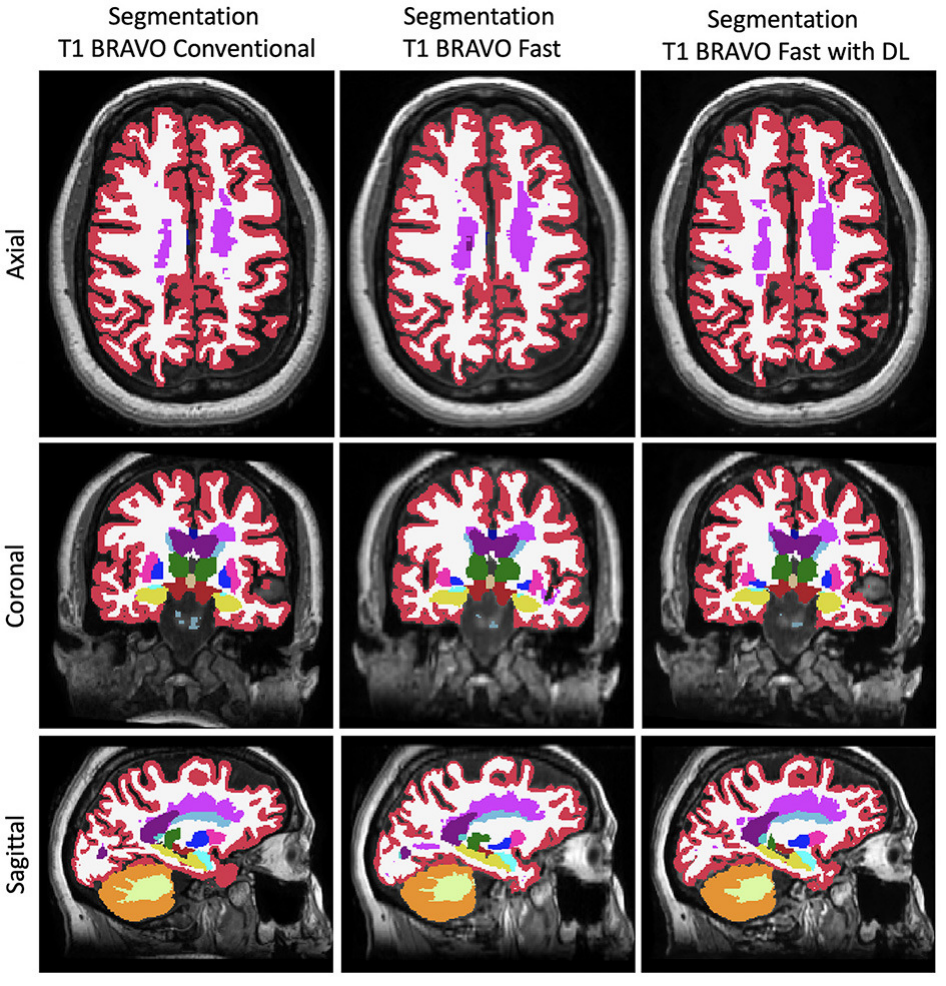
Representative FreeSurfer segmentation images. Slices in a similar position for the FreeSurfer segmentation derived from the three different methods to generate the images.

We compared the T1w volumetrics across the three MR acquisition methods: conventional scan, fast scan, and fast scan with DL (Figures 2, 3). For the T1w volumes, paired *t-*tests using the conventional scan as the benchmark indicated a true difference in mean volumes among these methods (Table 2). Using a threshold for multiple hypotheses testing, paired *t*-tests comparing fast scans against conventional scans were significant for NBV, NThV, NGMV, and NWMV (*p* = 0.0006, *p* = 0.01, *p* < 0.0001, and *p* < 0.0001, respectively). Likewise, paired *t*-tests comparing fast scans with DL against conventional scans were significant for NBV, NThV, NGMV, and NWMV (*p* < 0.0001, *p* = 0.002, *p* = <0.0001, and *p* < 0.0001, respectively). Compared to the conventional scans, the mean absolute difference (or percentage difference) for NBV, NThV, NGMV, and NWMV for fast scans with DL were 0.013 (−1.789%), 0.0020 (2.265%), 0.018 (−4.273%), and 0.0043 (1.478%), respectively. Finally, paired *t*-tests comparing fast scans with DL and fast scans were also significant for NBV, NThV, NGMV, and NWMV (*p* = 0.0004, *p* < 0.0001, *p* < 0.0001, and *p* < 0.0001, respectively).

**Table 2:** Paired differences for T1w volume measures across methods.

	**Mean of the differences ± SD**	***P*-values**	**Mean of the differences ±SD**	***P*-values**	**Mean of the Differences ±SD**	***P*-values**
	**Mean percentage difference**		**Mean percentage difference**		**Mean percentage difference**	
	**Fast vs. Conventional**	**Fast vs. Conventional**	**Fast with DL^b^ vs. Conventional**	**Fast with DL vs. Conventional**	**Fast with DL vs. Fast**	**Fast with DL vs. Fast**
NBV^a^	−0.007 ± 0.021 −0.905%	0.0006^*^	−0.013 ± 0.017 −1.789%	<0.0001^*^	−0.006 ± 0.015 −0.892%	0.0004^*^
NThV^a^	−0.0002 ± 0.0006 −1.641%	0.01^*^	0.0002 ± 0.0006 2.265%	0.002^*^	0.0004 ± 0.0005 3.971%	<0.0001^*^
NGMV^a^	−0.039 ± 0.012 −9.345%	<0.0001^*^	−0.018 ± 0.011 −4.273%	<0.0001^*^	0.022 ± 0.011 5.595%	<0.0001^*^
NWMV^a^	0.031 ± 0.018 10.874%	<0.0001^*^	0.004 ± 0.010 1.478%	<0.0001^*^	−0.026 ± 0.018 −8.475%	<0.0001^*^

a *NBV, normalized brain volume; NThV, normalized thalamic volume; NGMV, normalized gray matter volume; NWMV, normalized white matter volume. Normalized volumes have no unit*.

b *DL, deep learning approach applied to the fast scan*.

* *Indicated statistical significance meeting the multiple hypotheses testing threshold*.

While the T1w volumes on fast scan and fast scan with DL both had significant differences from conventional scans, the relative difference (or percentage difference) decreased from −9.3% (fast *vs*. conventional) to −4.3% (fast with DL vs. conventional) for normalized gray matter volume and from 10.9% (fast vs. conventional) to 1.5% (fast with DL vs. conventional) for normalized white matter volume. The relative difference marginally increased from −0.9% (fast vs. conventional) to −1.8% (fast with DL vs. conventional) for normalized brain volume and from −1.6% (fast vs. conventional) to 2.3% (fast with DL vs. conventional) for normalized thalamic volume.

### MRI-Clinical Correlations Comparisons

We next assessed the clinical applicability of the T1w volume metrics as acquired by the different methods again using the conventional scans as benchmarks. First, we examined the correlation between T1w volumetrics and physical and gait impairment based on the clinically relevant patient-reported outcome of PDDS (Table 3). The Wald chi-square test for comparing the coefficients between fast scans and the conventional scans were statistically significant only for NBV (*p* = 0.002) but not for NThV, NGMV, and NWMV, indicating a difference in the correlation between NBV (but not the other T1w volumetrics) and PDDS when comparing the fast scans to the conventional scans. Importantly, the coefficients comparing fast scans with DL and conventional scans were not statistically significant for NBV, NThV, NGMV, and NWMV, indicating no difference in the correlations between all T1w volumetrics and PDDS when comparing the fast scans with DL against the benchmark conventional scans.

**Table 3:** Linear mixed-effects model with conventional MRI scans as baseline and interaction with gait and physical impairment in multiple sclerosis as measured by PDDS.

	**Interaction between scan type and patient-reported outcomes^b^**	**Coefficient (SE)**	***P*-values for interaction term coefficients**
NBV^a^	Fast × PDDS	0.0031 (0.001)	0.002^*^
	FwDL × PDDS	0.0016 (0.001)	0.096
NThV^a^	Fast × PDDS	0.00007 (0.00003)	0.024
	FwDL × PDDS	0.000008 (0.00003)	0.790
NGMV^a^	Fast × PDDS	0.0010 (0.0006)	0.110
	FwDL × PDDS	0.0013 (0.0006)	0.039
NWMV^a^	Fast × PDDS	0.0020 (0.0008)	0.019
	FwDL × PDDS	0.0006 (0.0008)	0.460

a *NBV, normalized brain volume; NThV, normalized thalamic volume; NGMV, normalized gray matter volume; NWMV, normalized white matter volume. Normalized volumes have no unit*.

b *PDDS, Patient-determined disease steps; FwDL, Fast scan with deep learning*.

* *Indicated statistical significance meeting the multiple hypotheses testing threshold*.

Second, we examined the correlation between T1w volumetrics and the MS symptom burden based on the clinically relevant patient-reported outcome of MSRS-R (Table 4). There was no significant difference in the correlation between all T1w volumetrics (NBV, NThV, NGMV, and NWMV) and MSRS-R when comparing fast scans to the benchmark conventional scans. Likewise, we did not find statistical significance for the coefficients comparing fast scans with DL and the conventional scans for all T1w volumetrics, indicating no difference in the correlations between all T1w volumetrics and MSRS-R when comparing fast scans with DL against the benchmark scans.

**Table 4:** Linear mixed-effects model with conventional MRI scans as baseline and interaction with multiple sclerosis symptom burden as measured by MSRS-R.

	**Interaction between scan type and patient-reported outcomes^b^**	**Coefficient (SE)**	***P*-values for interaction term coefficients**
NBV^a^	Fast × MSRS-R	0.0007 (0.0003)	0.022
	FwDL × MSRS-R	0.0003 (0.0003)	0.297
NThV^a^	Fast × MSRS-R	0.00002 (0.00001)	0.025
	FwDL × MSRS-R	0.00002 (0.00001)	0.069
NGMV^a^	Fast × MSRS-R	0.0003 (0.0002)	0.102
	FwDL × MSRS-R	0.0002 (0.0002)	0.302
NWMV^a^	Fast × MSRS-R	0.0004 (0.0003)	0.181
	FwDL x MSRS-R	0.0001 (0.0003)	0.618

a *NBV, normalized brain volume; NThV, normalized thalamic volume; NGMV, normalized gray matter volume; NWMV, normalized white matter volume. Normalized volumes have no unit*.

b *MSRS-R, multiple sclerosis rating scale-revised; FwDL, fast scan with deep learning*.

* *Indicated statistical significance meeting the multiple hypotheses testing threshold*.

### Exploratory Analyses

**Figure 4:**
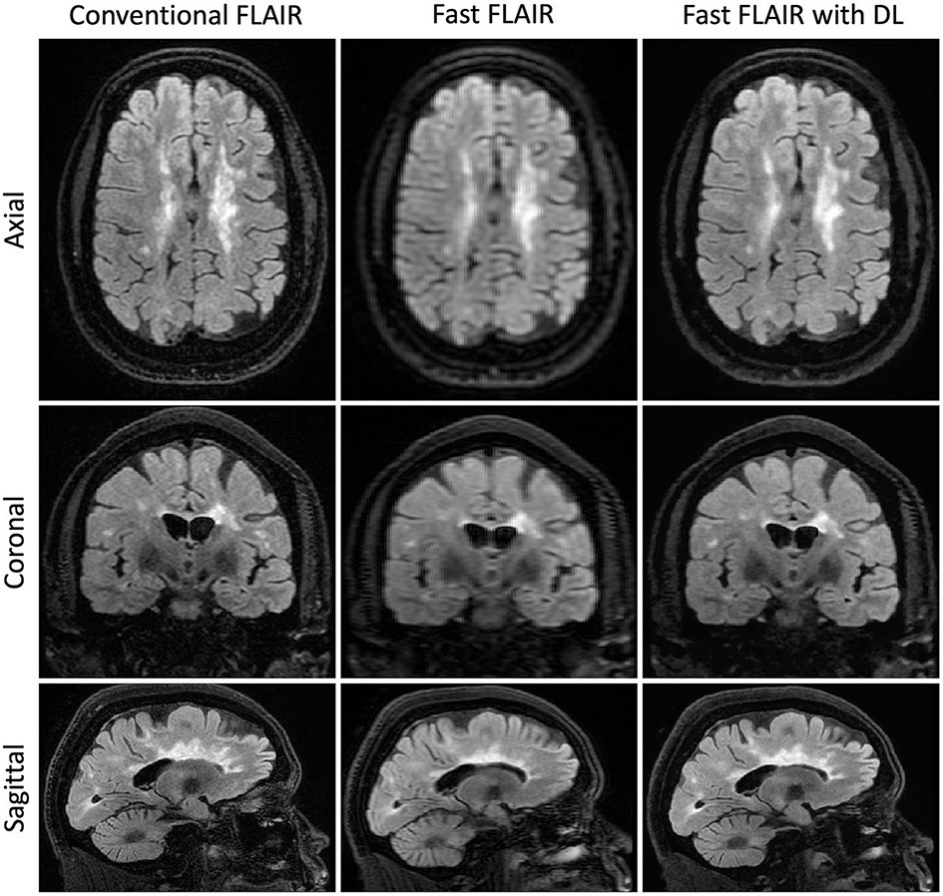
Representative T2FLAIR images. Slices in a similar position for the acquisitions using three different methods: Conventional T2 FLAIR (6:40 min total acquisition time, 256 × 224 × 240 matrix size), fast T2 FLAIR (1:13 min total acquisition time, 256 × 128 × 120 matrix size), and fast T2 FLAIR with DL (same acquisition time and matrix size as the fast T2 FLAIR). FLAIR is the T2-weighted Fluid-Attenuated Inversion Recovery sequence.

In a subset of the 30 scans in which we calculated T2 lesion volumes, there was no difference in pairwise comparisons across the three methods (Figure 4, Supplementary Table 1). Further, there was no difference in the correlation between the T2 lesion volume and either neurological outcome (MSRS-R or PDDS) when comparing fast scans with DL against the benchmark conventional scans (Supplementary Table 2).

## Discussion

We reported a deep learning model based on DBPN that improved the image quality of an accelerated T1w sequence acquired during routine clinical brain MR scans to the extent of preserving the correlation between the key T1w volumetrics and clinically relevant outcomes in PwMS. The T1w volumetrics (normalized brain volume, normalized thalamic volume, normalized gray matter volume, and normalized white matter volume) are all known to inform MS neurological outcomes (9–18) and are indeed inversely correlated with patient-reported neurological outcomes in this study (Supplementary Table 3). Here, the T1w (and T2w-FLAIR) fast images were acquired nearly 3 (and 6 times) faster than the conventional acquisitions (respectively). To our knowledge, this is the first report of DL application to improve the image quality of accelerated scans in clinical brain MR for MS and it has the potential for clinical applications in the routine care of PwMS.

Accelerating clinical MR acquisition time has direct clinical implications, particularly for conditions such as MS where routine disease monitoring using MR scans is the standard of care. Methods such as compressed sensing and parallel imaging aim to reconstruct higher quality images from a smaller amount of raw MR imaging data (28–30). However, the concern for clinical feasibility stems from the poor image quality and long reconstruction times (31). Deep learning methods began to address these issues by incorporating different types of CNN structures (3–6). These methods follow two similar steps: reducing scan acquisition time by under-sampling k-space in raw MR data and then reconstructing a higher-quality image using novel DL models. Contrasting feed-forward approaches, DBPN takes advantage of error feedback to self-correct at multiple layers of the neural network. Further, with its multiple stages of up- and down-sampling layers, it combines both local and global information into learning, and preserves the small details leading to more robust response compared to residual block-based models such as Enhanced Deep Residual Networks (EDSR), which focuses only on the local information (32). DBPN is a useful tool for improving image quality through noise elimination and sharpness-enhancement (8). Given it is not over-parameterized, a relatively modest sample size would be sufficient for training the model to generate reasonable performance without overfitting. Finally, it results in faster inference time during deployment without similar performance. To our knowledge, this specific application of DBPN to improve the quality of accelerated clinical brain MR scans for PwMS is novel.

In terms of the study design, we aimed to balance the need for sufficiently powered sample size for the held-out evaluations and the need to have sufficient training data to generate reasonable performance without overfitting. By tuning the model parameters (filter size, the number of filters, the number of the project units, etc.), it was feasible to build a model that performed well on a small training set. Empirically, we found that 15 cases for the current model setup (which were randomly selected) resulted in good performance in image improvement. The loss function metric, a measure of the difference between the model output and the ground truth image, decreased monotonically as validation set images were presented to the deep learning algorithm. This suggests that the model was not overfitted and could be generalizable. When visually assessing the image sharpness, particularly in the cerebral cortex, the performance of the validation set resembles the training set, again confirming that the model was not overfitted.

Although the T1w volumes computed from the fast scan and fast scan with DL both had significant differences when compared to the benchmark conventional scans (Table 2), the fast scan with DL reduced the mean absolute difference from the benchmark scans for normalized gray matter volume (from −9.345 to −4.273%) and normalized white matter volume (from 10.874 to 1.478%), while only marginally increased the mean absolute difference from the benchmark scans for normalized brain volume (from −0.905 to −1.789%) and normalized thalamic volume (from −1.641 to 2.265%) when compared to the fast scans. The current study cannot conclude whether the deep learning approach introduces brain region-specific improvement.

Despite the small T1w volumetric differences from the benchmark conventional approach, there was no significant difference in the correlations between all four T1w volumetrics and the two clinically relevant patient-reported outcomes of neurological disability and symptom burden (PDDS and MSRS-R) in the fast scans with DL against the benchmark conventional approach (Tables 3, 4). These findings indicate that the calculated volumetric differences between fast scans with DL and conventional approaches were not large enough to have clinical impact. Specifically, the correlation between the T1w MRI findings and real-world outcomes of gait and physical impairment (PDDS) as well as overall symptom burden (MSRS-R) were preserved in the fast scans with DL.

The difference in the parcellations derived from the fast and conventional images could explain the significant difference in the correlation between the normalized brain volume and PDDS (Table 3) as well as the borderline difference in the correlation between the normalized brain volume and MSRS-R (Table 4), though the latter association did not reach significance after correction for multiple testing. When calculating the normalized whole brain volume in the fast scans, the segmentation software likely performed less well when compared to the other T1w volumetrics. The coarse resolution of the fast scans might have made the delimitation of the whole brain edges more challenging.

Obtaining nearly equivalent image quality using shorter acquisition time improves patient comfort and satisfaction while reducing artifact introduced by involuntary motion that often manifests in the latter portion of a prolonged MR study. The increased MR study throughput would also enable efficient utilization of the MR resources, reducing unnecessary wait time and improving access to critical imaging for diagnostic and monitoring purposes, not only for PwMS but also for other patient populations.

There were limitations to our study. First, the study had a modest sample size, limiting the power of some of the statistical analyses (e.g., T2 lesion volume, see further discussion Supplementary Material). Second, the current study performed volumetric analysis on only the T1w images, while inclusion of the T2w-FLAIR contrasts in the processing pipeline might improve the volumetric estimations.

In summary, we demonstrated the clinical application of a deep learning model to improve the quality of accelerated T1w images in routine clinical brain MR scans for MS. Beyond further validation of this application in longitudinal studies (e.g., baseline T1w volumetrics informing long-term clinical outcomes), we anticipate future studies that test the ability of a DL model to replace gadolinium contrast for MRI with “virtual” contrast (33, 34).

## Data Availability Statement

The raw data supporting the conclusions of this article will be made available by the authors upon reasonable request to the corresponding author.

## Ethics Statement

The studies involving human participants were reviewed and approved by University of Pittsburgh. The patients/participants provided their written informed consent to participate in this study.

## Author Contributions

JM and ZX designed and conceptualized the study. MDa, SV, EW, JM, and ZX performed data collection. LW, TZ, and EG applied the deep learning model. TS, RP, MDe, CI, TI, JB-M, and F-CY performed imaging analysis. AM and RK performed statistical analysis. AM, TS, RP, and ZX drafted the manuscript. AM, TS, RP, F-CY, RK, JM, and ZX revised the manuscript. All authors approved the final manuscript.

## Funding

The research study was funded in part by a sponsored research agreement between University of Pittsburgh and Subtle Medical.

## Conflict of Interest

LW, TZ, and EG were employed by Subtle Medical, Inc. The authors also declare that the study received funding from Subtle Medical, Inc, through a sponsored research agreement between University of Pittsburgh and Subtle Medical. The funder had the following involvement in the study: application of the deep learning model to fast MRI scans, review, and revision of the article.

## Publisher's Note

All claims expressed in this article are solely those of the authors and do not necessarily represent those of their affiliated organizations, or those of the publisher, the editors and the reviewers. Any product that may be evaluated in this article, or claim that may be made by its manufacturer, is not guaranteed or endorsed by the publisher.
