# Supplementary material for: Applying Deep Learning to Accelerated Clinical Brain Magnetic Resonance Imaging for Multiple Sclerosis
Source: Front Neurol. 2021 Sep 27;12:685276. doi: 10.3389/fneur.2021.685276 (PMC8504490; doi:10.3389/fneur.2021.685276)
Supplement: Supplementary file 1 [file Data_Sheet_1.docx]

**Applying deep learning to accelerated clinical brain magnetic resonance imaging for multiple sclerosis**

Supplementary Material

**Mathematical Formula of the Repeated Measures Multivariate Linear Mixed -Effects Models.**

${volume}_{ij}=\beta_{0}+\beta_{1}{type}_{j} {+ \beta}_{2}{PDDS}_{i} {+ \beta}_{3} {type}_{j}*{PDDS}_{i}+ {age}_{i}+ {sex}_{i}+$
${nonhiswhite}_{i}{+ diseasedur}_{i}+ {RMS}_{i}+{stan}_{i}+ {high}_{i}$+ $\zeta_{i}$+$\epsilon_{ij}$

${volume}_{ij}=\beta_{0}+\beta_{1}{type}_{j} {+ \beta}_{2}{MSRS}_{i} {+ \beta}_{3} {type}_{j}*{MSRS}_{i}+ {age}_{i}+ {sex}_{i}+$
${nonhiswhite}_{i}{+ diseasedur}_{i}+ {RMS}_{i}+{stan}_{i}+ {high}_{i}$+ $\zeta_{i}$+$\epsilon_{ij}$

Where:

${volume}_{ij}=$brain volume measurement for subject i with scan type j

j = 1 Conventional Scan

j = 2 Fast scan

j = 3 Fast scan with DL

${PDDS}_{i} =$PDDS for subject i

${MSRS}_{i} =$MSRS-R for subject i

${age}_{i} =$age (at first scan) in years for subject i

${sex}_{i} =$sex (1 if female, 0 if male) for subject i

${nonhiswhite}_{i}=$race/ethnicity (1 if Non-Hispanic European descent, 0 if not)

${diseasedur}_{i} =$disease duration (in years) for subject i

${RMS}_{i} =$clinical type of MS (1 if relapsing clinical type, 0 if not) for subject i

${stan}_{i}=$standard-efficacy treatment status (1 if standard treatment, 0 if not)

${high}_{i}=$high-efficacy treatment status (1 if standard treatment, 0 if not)

$\zeta_{i} =$random intercept for subject i

$\epsilon_{ij} =$error term for subject i with scan type j

**Exploratory Analysis of T2 Lesion Volume**

In exploratory analysis examining T2 lesion volume, we first performed paired t-tests to compare the three types of MR acquisition (conventional, fast, fast with DL). There were no significant differences in pairwise comparisons across the three MR acquisition methods (**Supplementary Table 1**). Using the conventional scan as the benchmark, the mean absolute difference (and percentage difference) marginally increased from 552.72 mm^3^ (-7.9%, fast *vs.* conventional) to 591.65 mm^3^ (-8.4%, fast with DL *vs.* conventional). The expected sample size needed to achieve 80% power at a significance level of 0.05 for comparing fast scans to conventional scans, fast scans with DL to conventional scans, and fast scans with DL to fast scans are 97, 210, and 3628 subjects, respectively. Thus, with our sample size of 30 scans for T2 lesion volume we achieved 33%, 17%, and 5% power, respectively.

Using the repeated measures multivariate linear mixed-effects model and Wald chi-square tests, we examined the correlation of the T2 lesion volume with the clinically relevant patient-reported outcome of MS symptom burden (MSRS-R) and physical / gait impairment (PDDS) by computing the significance of the interaction coefficients (**Supplementary Table 2**). There was no significant difference for the coefficient comparing fast scans and the conventional scans *or* the coefficient comparing fast scans with DL and the conventional scans for either set of outcome correlations. These results indicated no difference in the correlation between the T2 lesion volume and neurological outcome (MSRS-R or PDDS) when comparing fast scans with DL against the benchmark conventional scans.

The subgroup analysis of T2 lesion volume quantification relied on a process of manual correction by two initial raters and a supervising rater. This labor-intensive process limited the sample size for the T2 analysis.

**Supplementary Table 1.** Paired differences for T2 lesion volume across methods.

|  | **Fast**  **v.**  **Conventional**  **Mean of the Differences +SD**  **Mean Percentage Difference** | **Fast**  **v.**  **Conventional**  **P-values** | **Fast with DL ^1^**  **v.**  **Conventional**  **Mean of the Differences +SD**  **Mean**  **Percentage Difference** | **Fast with DL v.**  **Conventional**  **P-values** | **Fast with DL**  **v.**  **Fast**  **Mean of the Differences +SD**  **Mean**  **Percentage Difference** | **Fast with DL v.**  **Fast**  **P-values** |
| --- | --- | --- | --- | --- | --- | --- |
| T2 Lesion Volume (mm^3^) | -748.72 ± 2603.51  -7.893% | 0.133 | -591.65 ± 3039.31  -8.449% | 0.295 | 120.82 ± 2596.77  -0.604% | 0.804 |

Note:

- - - 1. DL, deep learning approach applied to the fast scan.

**Supplementary Table 2.** Linear mixed-effects model for T2 lesion volume with conventional MRI scans as the benchmark and interaction with clinically relevant patient-reported outcomes

|  | **Interaction between Scan Type and Patient-Reported Outcomes** ^1^ | **Coefficient (SE)** | **P-values for Interaction Term Coefficients** ^2^ |
| --- | --- | --- | --- |
| T2 Lesion Volume (mm^3^) | Fast x PDDS | -316.68 (266.62) | 0.235 |
|  | FwDL x PDDS | -141.19 (262.68) | 0.591 |
|  | Fast x MSRS-R | -104.68 (92.18) | 0.256 |
|  | FwDL x MSRS-R | -9.28 (90.53) | 0.918 |

Note:

PDDS: Patient-determined disease steps; MSRS-R: Multiple Sclerosis Rating Scale-Revised; FwDL: Fast scan with deep learning

**Supplementary Table 3**. Pearson Correlations Among T1 Volume Measures (acquired using conventional MRI scans) and Patient-Reported Outcomes

|  | PDDS ^2^ | MSRS-R ^2^ |
| --- | --- | --- |
| NBV ^1^ | -0.466 * | -0.320 * |
| NThV ^1^ | -0.487 * | -0.365 * |
| NGMV ^1^ | -0.541 * | -0.410 * |
| NWMV ^1^ | -0.316 * | -0.165 |

1. NBV: normalized brain volume; NThV: normalized thalamic volume; NGMV: normalized gray matter volume; NWMV: normalized white matter volume. Normalized volumes have no unit.

2. PDDS: Patient-determined disease steps

3. MSRS-R: Multiple Sclerosis Rating Scale-Revised

4. * indicated statistical significance of the correlation meeting the multiple hypotheses testing threshold.
